# Phosphorylation of MET Is Upregulated in Metastatic Sites of Renal Cell Carcinoma: Possible Role of MET and Hepatocyte Growth Factor Activation-Targeted Combined Therapy

**DOI:** 10.3390/biomedicines13040811

**Published:** 2025-03-28

**Authors:** Takahiro Akioka, Shoichi Kimura, Yuichi Katayama, Masato Fujii, Takumi Kiwaki, Makiko Kawaguchi, Tsuyoshi Fukushima, Yuichiro Sato, Shoichiro Mukai, Toshiyuki Kamoto, Atsuro Sawada

**Affiliations:** 1 Department of Urology, Faculty of Medicine, University of Miyazaki, Miyazaki 889-1692, Japan; takahiro_akioka@med.miyazaki-u.ac.jp (T.A.); yuuichi_katayama@med.miyazaki-u.ac.jp (Y.K.);; 2 Section of Oncopathology and Morphological Pathology, Department of Pathology, Faculty of Medicine, University of Miyazaki, Miyazaki 889-1692, Japan

**Keywords:** renal cell carcinoma, metastasis, MET, HGF

## Abstract

**Background:** Increased expression of MET and hepatocyte growth factor (HGF)-related molecules has been positively correlated with poor prognosis in renal cell carcinoma (RCC). In the current study, the expression and phosphorylation of MET in metastatic RCC (mRCC) are determined by immunohistochemistry, and the therapeutic effect of MET and HGF activation-targeting agents for RCC cell lines is analyzed. **Methods:** Immunohistochemistry was performed for 76 formalin-fixed paraffin-embedded specimens (primary tumor: 32, metastatic site: 44). The therapeutic effect of capmatinib (MET-I) and SRI-31215 (inhibitor of HGF-activating proteases: HGFA-I) was determined based on the inhibition of MET phosphorylation, cell proliferation, and cell migration in 786-O and caki-1 cell lines. **Results:** Increased expression and phosphorylation of MET were observed in both primary tumor and metastatic sites; however, phosphorylation was significantly upregulated in metastatic sites (*p* = 0.0001). In an assay of RCC cell lines, the strongest inhibition of MET phosphorylation, cell proliferation, and migration was confirmed with the combined used of MET-I and HGFA-I. **Conclusions:** Phosphorylation of MET was significantly upregulated in metastasis, which suggested the importance of downregulation in the treatment of mRCC. Our findings suggest that dual inhibition of MET and HGF activation may offer a promising strategy for mRCC treatment, warranting further clinical validation.

## 1. Introduction

Hepatocyte growth factor (HGF) is a multifunctional growth factor known to induce the progression of various cancers through the specific tyrosine kinase receptor MET [1,2]. In cancer, HGF is reported to be secreted mainly from cancer-associated fibroblasts (CAFs) in the paracrine fashion [3,4], though some cancers have the potential to express HGF in the autocrine style [5]. HGF is primarily secreted as an inactive pro-form (pro-HGF), and proteolytical activation by a specific HGF-activating protease, including HGF activator (HGFA), hepsin, and matriptase, is necessary to gain the biological function [2]. Binding of active HGF to MET induces dimerization of MET, and phosphorylation occurs. As a result, a downstream intracellular signaling pathway is activated. Activation of the HGF/MET signaling axis promotes cancer cell proliferation, anti-apoptosis, motility, invasiveness, and resistance to therapeutic agents [6,7]. In addition, a correlation between an increased expression of MET in cancer and a poor prognosis has been reported in various human cancers [8,9].

Renal cell carcinoma (RCC), the most common renal malignant tumor in adults, has been reported to result in metastasis in approximately 30% of patients [10]. As major therapeutic agents for metastasis, a combination of immune checkpoint inhibitors and tyrosine kinase inhibitors (TKIs), which target receptors of growth factors, including vascular endothelial growth factor receptor (VEGFR), platelet-derived growth factor receptor, fibroblast growth factor receptor, and MET, is widely used [10,11,12,13,14]. In RCC, the activating mutation of MET in hereditary papillary RCC provides well-known evidence, and MET expression induced by hypoxia-inducible factors (HIFs) in clear-cell RCC (ccRCC) is also common evidence [15,16]. Indeed, the increased expression of HGF, MET, and enhanced activation of HGF have been seen in ccRCC [13,17]. In addition, increased expression of MET and hepsin, a specific activator of pro-HGF, has been positively correlated with a poor prognosis in ccRCC [17]. Therefore, HGF-dependent MET activation has a significant role in the progression of RCCs [17]. In the treatment of metastatic RCC (mRCC), cabozantinib, a potent TKI targeting VEGFR and MET, has demonstrated an acceptable therapeutic effect [18]. In the previous study, we evaluated the expression of MET and matriptase in the primary tumor and bone metastasis of RCC by using immunohistochemistry (IHC) [19]. A higher expression of both molecules was observed in bone metastasis compared with the primary tumor (MET: 86 vs. 47%, matriptase: 100 vs. 35%). Because the ubiquitous expression of HGF has been reported, upregulation of MET phosphorylation due to matriptase-induced hyperactivation of pro-HGF was suggested.

In this study, we found increased phosphorylation of MET in metastatic sites compared with primary tumors when evaluating the expression and phosphorylation of MET in mRCC by performing immunohistochemistry of surgically resected specimens. Then, we analyzed the significance of the inhibition of the HGF/MET signaling axis for RCC cell lines with the combination of an MET inhibitor (MET-I) and HGF-activator inhibitor (HGFA-I).

## 2. Materials and Methods

### 2.1. Clinicopathological Study Cohort

In this study, clinical data were obtained from clinical records retrospectively. The tumor specimens were from paraffin-embedded blocks. We selected 31 patients who received both nephrectomy and metastasectomy (including biopsy) for RCC between April 2012 and November 2021, including 32 primary RCC and 44 metastatic specimens. Paraffin-embedded tissue sections of the resected tumor tissues were processed for H&E staining and immunohistochemical staining. The study protocol was in accordance with the revised Declaration of Helsinki of 1983 and approved by the Institutional Review Board of the Faculty of Medicine, University of Miyazaki (approval number: O-0132).

### 2.2. Antibodies and Reagents

Antibodies specific to phospho-MET (Y1234/1235), MET, ST14/Matriptase, phospho-Akt (Ser473), Akt, phospho-p44/42MAPK (Thr202/Tyr204), p44/42MAPK, phospho-SAPK/JNK (Thr183/185), SAPK/JNK, and alpha tubulin were purchased from Cell Signaling Technology (Danvers, MA, USA), and the anti-human hepsin antibody and anti-human HGF antibody were purchased from Abcam (Cambridge, UK).

An antibody against c-MET (Y1235 phosphorylated) purchased from Immuno-Biological Laboratories (Gunma, Japan), an antibody against Met purchased from Santa Cruz Biotechnology (Santa Cruz, CA, USA), and an antibody against CD10 purchased from Leica Biosystems (Nussloch, Germany) were used for immunohistochemistry. Recombinant human pro-HGF was sourced from R&D systems (Minneapolis, MN, USA). Recombinant human HGF was purchased from PeproTech (Cranbury, NJ, USA). Capmatinib and SRI-31215 were obtained from MedChemExpress (Monmouth Junction, NJ, USA).

### 2.3. Cell Culture

Human RCC cell lines 786-O and caki-1 were purchased from the American Type Culture Collection (Manassas, VA, USA). We cultured 786-O in RPMI 1640 (Gibco/Life Technologies, Carlsbad, CA, USA) containing 10% fetal bovine serum (FBS, Gibco/Life Technologies, Carlsbad, CA, USA) at 37 °C in a humidified atmosphere of 5% CO_2_. Caki-1 was cultured in EMEM (Wako, Osaka, Japan) containing 10% FBS at 37 °C in a humidified atmosphere of 5% CO_2_.

### 2.4. RNA Extraction

Total RNA was extracted using a PureLink RNA Mini Kit (Invitrogen, Carlsbad, CA, USA) according to the manufacturer’s directions. mRNA was reverse transcribed using a PrimeScript FAST RT reagent kit with gDNA Eraser (Takara, Shiga, Japan) according to the manufacturer’s directions.

### 2.5. Real-Time Quantitative PCR

We performed real-time PCR analyses with a Thermal Cycler Dice Real Time System II (Takara, Shiga, Japan). Reaction mixtures (25 µL) containing 2 µL of cDNA template, 1 µL each of sense and anti-sense primers, and 1×SYBR Premix Ex Taq II (Takara, Shiga, Japan) were amplified as follows: held at 95 °C for 30 s and 40 cycles at 95 °C for 5 s, 60 °C for 30 s, and final dissociation stage (95 °C for 15 s, 60 °C for 30 s, and 95 °C for 15 s). The results were evaluated by Thermal Cycler Dice Real Time System software program version 5.11 (Takara, Shiga, Japan). The delta–delta Ct (ΔΔCt) algorithm was used to analyze the relative changes in gene expression. Experiments were repeated in triplicate, and GAPDH was used as an internal control. The primers were as follows: GAPDH forward, 5′-GCACCGTCAAGGCTGAGAAC-3′ and reverse, 5′-TGGTGAAGACGCCAGTGGA-3′; MET forward, 5′-TCCCATCAACAGGACTACACACTT-3′ and reverse, 5′-GCTGCAGGTATAGGCAGTGACAA-3′; HGF forward, 5′-GTTCAATGTGGGACAAGAACATGG-3′ and reverse, 5′-GGATTTCGGCAGTAATTCTCATTCA-3′; ST14 forward, 5′-GAGCAAGGGCAACCCTGAGT-3′ and reverse, 5′-CCCAACAACACGAGCCTGTC-3′; hepsin forward, 5′-GTCTGCAATGGCGCTGACTTCT-3′ and reverse, 5′-TCCGAGAGATGCTGTCCTCACA-3′.

### 2.6. Protein Extraction and Immunoblot Analysis

Cells were washed twice with ice-cold phosphate-buffered saline followed by incubation with the RIPA lysis buffer (Thermo Fisher Scientific Waltham, MA, USA). The degenerated cells were scraped and collected into microcentrifuge tubes and centrifuged at 13,000 rpm and 4 °C for 15 min. The extracted protein was used for immunoblot analyses. The reaction samples were mixed with sodium dodecyl sulfate–polyacrylamide gel electrophoresis (SDS-PAGE) sample buffer. The mixture was heated for 15 min at 75 °C. SDS-PAGE was performed under reducing conditions using 4–12% gradient gels. After electrophoresis, the sample proteins were transferred electrophoretically to iBlot 2 Transfer Stacks PVDF membranes (Thermo Fisher Scientific) using an iBlot 2 Gel Transfer Device. After blocking the nonspecific binding site with PDVF Blocking Reagent (TOYOBO, Tokyo, Japan), the membranes were incubated with primary antibody in Can Get Signal Solution 1 (TOYOBO) at 4 °C overnight followed by four washes with the buffer and incubation with peroxidase-conjugated secondary antibody diluted in Can Get Signal Solution 2 (TOYOBO) at room temperature. The labeled proteins were visualized with ECL Prime Western Blotting Detection Reagents (Global Life Sciences Solutions, Richmond, UK).

### 2.7. Cell Proliferation Assay: Dual Inhibition by MET-I and HGFA-I

In a 96-well plate, 5 × 10^3^ cells were seeded in 100 μL of medium and incubated for 24 h. The cells were then treated under conditions with or without MET-I and HGFA-I. Subsequently, pro-HGF was added at a concentration of 100 ng/mL. After a further incubation of 48 h, cell viability was assessed using a 3-(4,5-dimethylthiazol-2-yl)-5-(3-carboxymethoxyphenyl)-2-(4-sulfophenyl)-2H-tetrazolium (MTS) assay. We added 20 μL of CellTiter 96 Aqueous One Solution (Promega) to each well. The plate was then incubated for an additional 2 h at 37 °C, after which the absorbance of each well was measured at 490 nm. Six independent experiments were performed for statistical calculation.

### 2.8. Wound-Healing Assay: Dual Inhibition by MET-I and HGFA-I

We seeded 786-O and caki-1 cells on a 24-well plate. Cells were cultured by creating a confluent monolayer, and then a scratch was made. A scratch/wound with clear edges was created across the width of the well with a 200 μL pipette tip and was washed twice with phosphate-buffered saline to remove debris or detached cells. The cells were then treated under conditions with or without MET-I and HGFA-I. Subsequently, pro-HGF was added at a concentration of 100 ng/mL. The wound field area was measured at the start and at 9 h for 786-O and at the start and 48 h for caki-1. The wound field area was measured with ImageJ software version 1.54g (NIH). The percent closure was determined as follows: percent closure (%) = non-cell surface area of 9 h or 48 h/non-cell surface area of 0 h × 100. Four independent experiments were performed for statistical calculation. The closure rates of each cell line were compared using one-way ANOVA with Tukey’s multiple-comparison test.

### 2.9. Immunohistochemistry and Analysis

Formalin-fixed paraffin-embedded sections were prepared in the ordinary way. The specimens of bone metastasis were subjected to a decalcification procedure with 10% ethylenediamine-tetra-acetic acid (pH 7.2) for 24 h. For immunohistochemistry, sections were processed for antigen retrieval (microwaved in 10 mM citrate buffer, pH 6.0 for 10 min), followed by treatment with 3% H_2_O_2_ in methanol for 10 min and washing in tris-buffered saline (TBS) twice. After blocking in 3% bovine serum albumin and 5% goat serum in phosphate-buffered saline for 2 h at room temperature, the sections were incubated with primary antibodies at 4 °C overnight. Sections were then washed in TBS and incubated with Envison-labeled polymer reagent (DAKO, Santa Clara, CA, USA) for 30 min at room temperature. Sections were exposed to nickel, cobalt-3, and 3-diaminobenzidine (Immunopure Metal Enhanced DAB Substrate Kit; Piece, Rockford, IL, USA) and counterstained with hematoxylin. The procedure of MET phosphorylation and immunostaining has been described previously [20].

The staining intensity of the immunoreaction was judged by the percentage of RCC cells in which the cell membranes were stained with or without cytoplasm (e.g., if 50 out of 100 cells were stained, staining was 50%): staining of >10%, positive; <10% or weak staining, negative. Threshold positivity was defined by referring to the previous study [21].

### 2.10. Statistical Analysis

SPSS statistics version 25.0 (SPSS, Chicago, IL, USA) was used to assess statistical parameters. Pearson’s Chi-square test was used to assess the significance of correlation between the expression or phosphorylation of MET and metastasis. One-way ANOVA with Tukey’s multiple-comparison test was used to assess the significance of differences between individual groups in the proliferation assay and wound-healing assay. Differences were considered statistically significant when the *p*-value was <0.05.

## 3. Results

### 3.1. Expression of MET and the Phosphorylation in Metastatic RCC

**Figure 1:**
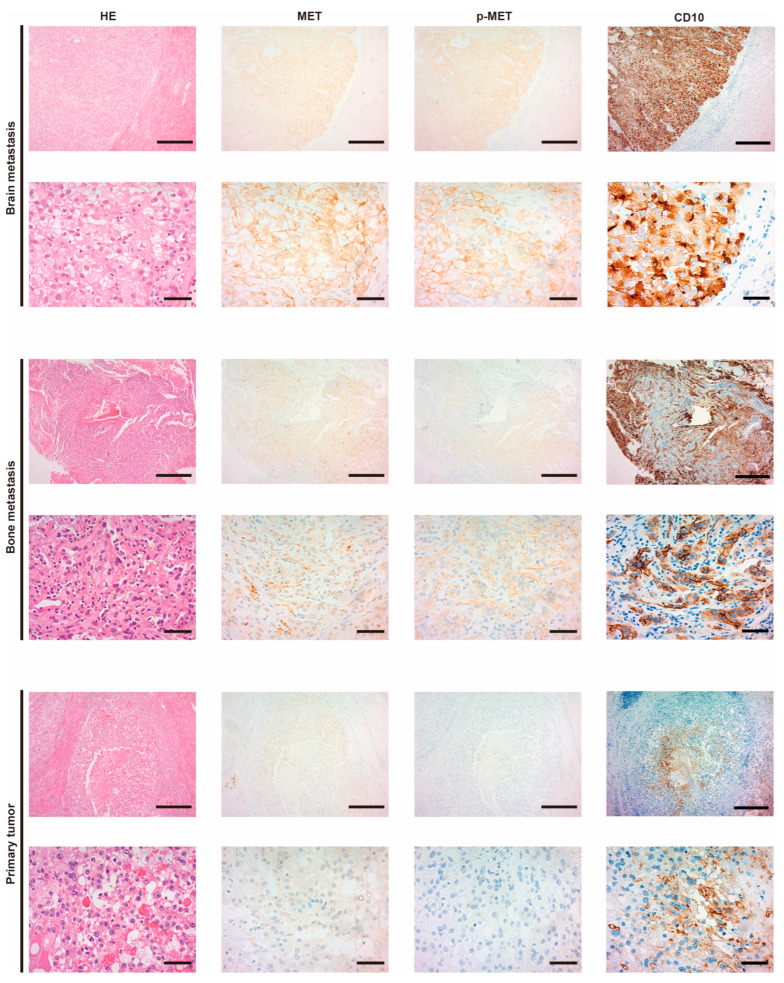
Expression of MET and the phosphorylation in RCC, according to immunohistochemical analysis.

Patient characteristics are shown in Table 1. The representative appearance of immunohistochemistry is shown in Figure 1. Positive staining of MET and p-MET, which was defined as membranous staining with or without cytoplasmic staining, was observed in cancer cells. The two upper horizonal lines are brain metastasis (upper line: low magnification, lower line: high magnification). The strongest expression and phosphorylation of MET were observed in brain metastasis (judged as positive). The two middle lines are bone metastasis, which show an increased expression of MET and phosphorylation (judged as positive). The two lower lines are the primary tumor. Expression of MET was very weak (judged as negative) and phosphorylation was not observed (negative). Increased expression of MET was observed in 29 of 31 (93.5%), and increased phosphorylation of MET was observed in 4 of 31 (12.9%) at the primary tumor. In metastasis, MET expression was observed in 37 of 44 (84.1%), and phosphorylation of MET was upregulated in 31 of 44 (70.5%). Phosphorylation of MET was thus significantly upregulated in metastasis (Table 2, *p* = 0.0001). No statistical correlation was observed in pathological T staging. In addition, no apparent correlation between MET expression, MET phosphorylation, and prognosis was observed (Supplemental Figures S1–S3). In metastasis (Table 3), expression of MET was increased in subcutaneous (5/5, 100%), lymph node (5/5, 100%), lung (12/14, 86%), and bone (6/8, 75%) samples. MET phosphorylation was particularly evident in subcutaneous (5/5, 100%) and bone metastasis (6/8, 75%) samples. Details of the comparative immunoreactivity of MET and p-MET are shown in Supplemental Tables S1–S3.

Representative histological findings are shown. The two upper horizonal lines are brain metastasis (upper line: low magnification, lower line: high magnification), the two middle lines are bone metastasis (upper line: low magnification, lower line: high magnification), and the two lower lines are the primary tumor (upper line: low magnification, lower line: high magnification). Vertical columns are, from left to right, hematoxylin–eosin (HE) staining, immunohistochemical staining of MET, and phosphorylation of MET (p-MET) and CD10. CD10 was used as biomarker of RCC. Brain metastasis was judged as positive in both MET and p-MET (strongly positive). Bone metastasis was also judged as positive in both MET and p-MET. The primary tumor was judged as negative in both MET and p-MET. Scale bar = 500 μm (low magnification), 50 μm (high magnification).

### 3.2. Expression of HGF/MET Signaling-Related Molecules in RCC Cell Lines

**Figure 2:**
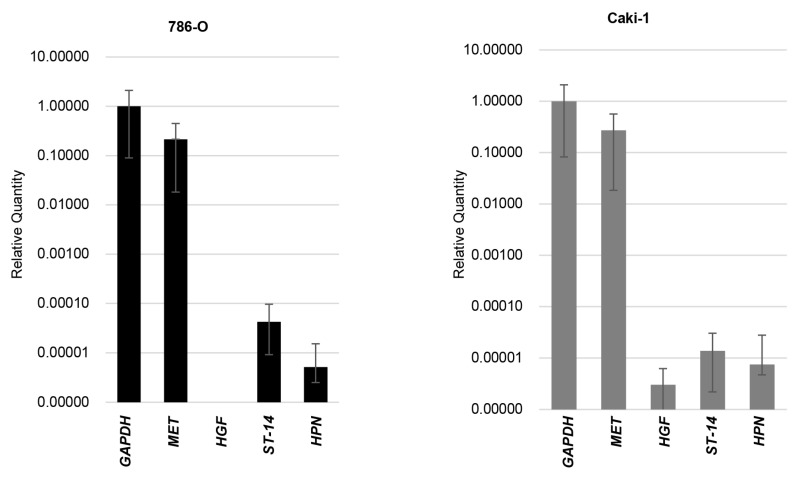
Expression of HGF-related molecules in RCC cell lines.

Initially, we evaluated the expression of MET, HGF, HPN (encoded hepsin: activator of pro-HGF), and ST-14 (encoded matriptase: activator of pro-HGF) in 786-O and caki-1 cells by real-time quantitative PCR (Figure 2). A strong expression of MET was observed in both cell lines, and expression of HPN and ST-14 was also confirmed in both cell lines. Expression of HGF was absent in 786-O, and weak expression was observed in caki-1.

The expression of *MET*, *HGF*, *ST-14*, and *HPN* was analyzed by real time RT-qPCR.

### 3.3. Inhibition of MET Phosphorylation by MET-I and HGFA-I

For the inhibition of MET phosphorylation, we used capmatinib (MET-I), a highly selective TKI of MET, and SRI-31215 (HGFA-I), a synthetic inhibitor of hepsin and matriptase, to inhibit the activation of pro-HGF. Efficacy was evaluated in the presence of pro-HGF (100 ng/mL).

**Figure 3:**
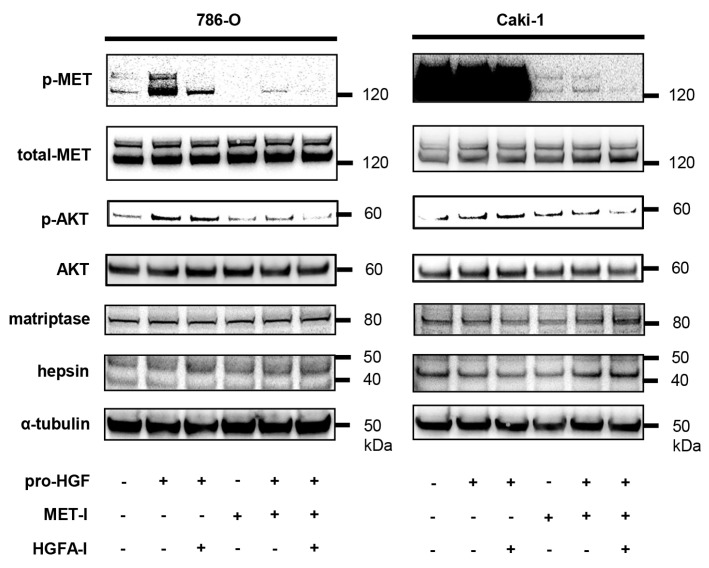
Inhibition of MET phosphorylation by MET-I and HGFA-I.

HGF-induced phosphorylation of MET was confirmed in 786-O (phosphorylation was faintly observed in the absence of HGF), and HGF-independent phosphorylation of MET was observed in caki-1 (Figure 3). In 786-O, a single use of MET-I inhibited MET phosphorylation and a single use of HGFA-I inhibited MET phosphorylation slightly; however, phosphorylation remained with both drugs. The strongest inhibition was observed with a combination of MET-I and HGFA-I. In both cell lines, a single use of MET-I inhibited MET phosphorylation, but pro-HGF caused MET phosphorylation in the presence of MET-I. A combination of MET-I and HGFA-I inhibited MET phosphorylation completely in the presence of pro-HGF. We also analyzed the phosphorylation of AKT and MAPK, which are major downstream signaling proteins mediating the signaling axis of receptor tyrosine kinase. A tendency for a downregulation of AKT phosphorylation was observed in both cell lines; however, no apparent changes were observed in MAPK (Supplemental Figure S4).

The results of the immunoblot analysis are shown in Figure 3. We pre-cultured 786-O and caki-1 cells in FBS-free medium for 24 h. The cells were then treated with each agent (final concentration of 2 nM for MET-I and 40 µM for HGFA-I in 786-O, 4 nM for MET-I and 20 µM for HGFA-I in caki-1) at 37 °C for 1 h, followed by the addition of recombinant pro-HGF (100 ng/mL). After incubation at 37 °C for 3 h, the proteins were extracted. The phosphorylation of MET, expression of total MET, and phosphorylation of AKT, AKT, matriptase, hepsin, and α-tubulin were determined by immunoblot analysis.

### 3.4. Inhibition of RCC Cell Proliferation by MET-I and HGFA-I

**Figure 4:**
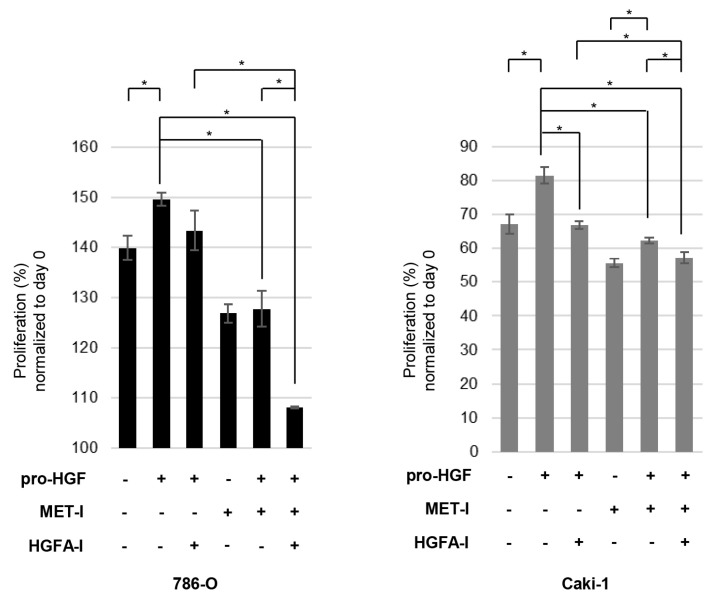
Effect of inhibition of MET and HGF activation on RCC cell proliferation (* *p* < 0.05).

Next, we analyzed the effect on cell proliferation. Proliferation of both cell lines was significantly upregulated in the presence of HGF (Figure 4). In Figure 3, caki-1 showed auto-phosphorylation of MET in the absence of HGF; however, additional HGF upregulated proliferation. A single use of HGFA-I reduced the proliferation of caki-1 in the presence of HGF. In 786-O, a single use of HGFA-I slightly reduced proliferation in the presence of HGF; however, significance was not observed. A significant reduction was observed with the single use of MET-I in the presence and absence of HGF in both cell lines. In caki-1, the therapeutic effect of MET-I was reduced in the presence of HGF compared with its absence. The strongest inhibition was observed for a combination of MET-I and HGFA-I in both cell lines.

In a 96-well plate, 5 × 10^3^ cells (786-O and caki-1) were seeded in 100 μL of medium and incubated for 24 h. The cells were then treated under conditions with or without MET-I (20 μM) and HGFA-I (20 μM). Subsequently, pro-HGF was added at a concentration of 100 ng/mL. After a further incubation of 48 h, cell viability was assessed using a 3-(4,5-dimethylthiazol-2-yl)-5-(3-carboxymethoxyphenyl)-2-(4-sulfophenyl)-2H-tetrazolium (MTS) assay. We added 20 μL of CellTiter 96 Aqueous One Solution (Promega) to each well. The plate was then incubated for an additional 2 h at 37 °C, after which the absorbance of each well was measured at 490 nm. Six independent experiments were performed for statistical calculation. 

### 3.5. Inhibition of RCC Cell Motility by MET-I and HGFA-I

**Figure 5:**
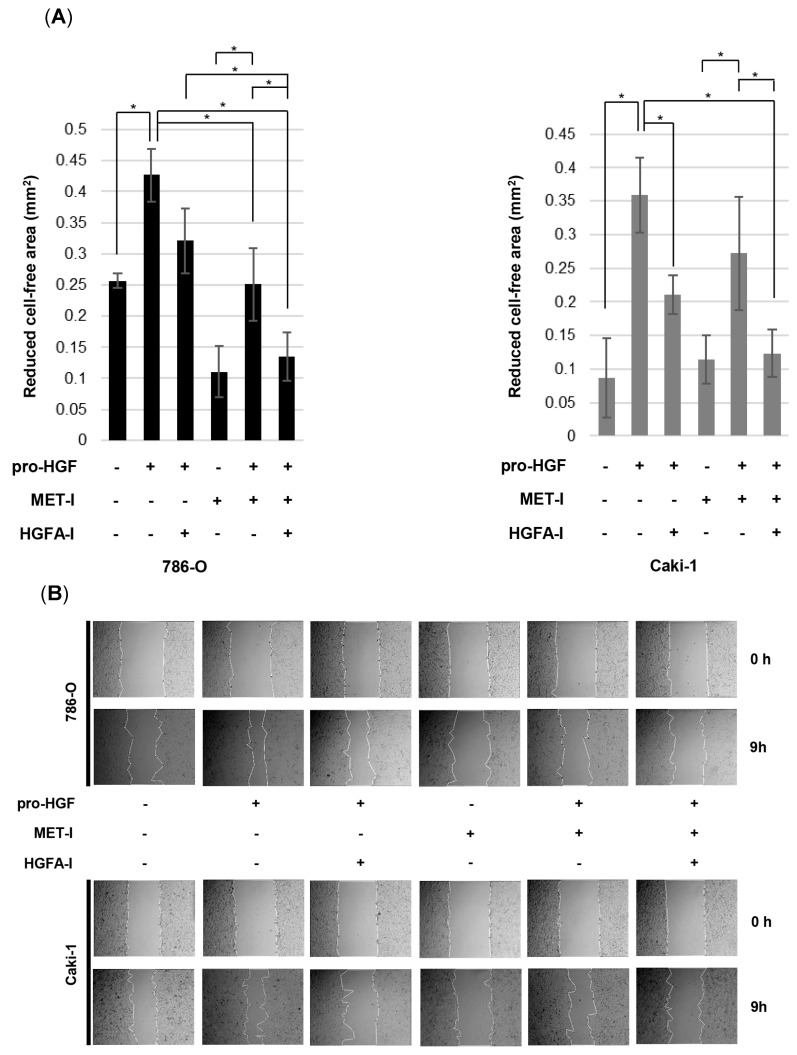
Effect of inhibition of MET and HGF activation on RCC cell migration. (**A**) The extent of cell movement was calculated in the indicated periods and is displayed as the mean ± SE of four independent experiments. (**B**) Photomicrographs of 786-O and Caki-1 cells. * *p* < 0.05.

The inhibitory effect of MET-I and HGFA-I on cell migration was determined by a monolayer wound-healing assay (Figure 5A,B). Cell motility was significantly upregulated in the presence of HGF compared with its absence. A significant therapeutic effect was observed in caki-1 with the single use of HGFA-I in the presence of HGF. Motility of 786-O was also downregulated by HGFA-I; however, significance was not observed (*p* = 0.069). Although MET-I significantly inhibited the motility of 786-O, no significant effect was observed for caki-1 in the absence of HGF. Of interest, additional HGF induced an upregulation of cell motility even in the presence of MET-I, suggesting HGF-induced resistance for MET-I. Finally, resistance was overcome by the combination of MET-I and HGFA-I in both cell lines.

We seeded 786-O and caki-1 cells on a 24-well plate. Cells were cultured by creating a confluent monolayer, and then a scratch was made. A scratch/wound with clear edges was created across the width of the well with a 200 μL pipette tip and was washed twice with phosphate-buffered saline to remove debris or detached cells. The cells were then treated under conditions with or without MET-I (20 μM for 786-O and 10 µM for Caki-1) and HGFA-I (10 μM). Subsequently, pro-HGF was added at a concentration of 100 ng/mL. The wound field area was measured at the start (0 h) and 9 h (9 h) for 786-O and 48 h (48 h) for caki-1. The percent closure was determined as follows: percent closure (%) = non-cell surface area of 9 h or 48 h/non-cell surface area of 0 h × 100. Four independent experiments were performed for statistical calculation. 

## 4. Discussion

In this study, we investigated the expression and phosphorylation of MET in surgically resected specimens from patients with mRCC by immunohistochemistry. Increased expression of MET was observed in both the primary tumor and metastasis (93.5 and 84.1%). However, phosphorylation was not evident in the primary tumor (12.9%) while it was upregulated in metastasis (70.5%). In vitro analysis using two major RCC cell lines revealed that the downregulation of MET phosphorylation significantly reduced RCC cell proliferation and migration. These findings suggested the importance of MET inactivation in the treatment of mRCC patients. In the analysis of IHC for metastasis, MET phosphorylation was upregulated in bone metastasis (Table 3). Increased expression of MET and matriptase in RCC bone metastasis was reported previously [19]. As mentioned above, higher expression of both molecules was observed in bone metastasis compared with the primary tumor. To explain the current IHC results, an upregulation of MET phosphorylation in metastasis (including bone) due to matriptase-induced hyperactivation of pro-HGF is suggested. In addition, the significant therapeutic effect of cabozantinib, which targets MET, VEGFR2, and AXL, for bone metastasis in patients with RCC, prostate cancer, and breast cancer also indicates the importance of MET inhibition in the treatment of bone metastasis [10,22,23].

Increased expression of MET has been reported to correlate with a poor prognosis in various cancers [2,9]. In bladder cancer, a correlation between the phosphorylation of MET and a poor prognosis was also reported [20,24]. However, the apparent correlation between MET expression, MET phosphorylation, and the prognosis was not observed in our study (Supplemental Figures S2–S4). This study has limitations, however, because the evaluation was performed on a small number of clinical samples, and patient selection bias was a factor (e.g., only patients who could afford the surgery were selected). Further study on a larger cohort is necessary to accurately clarify the correlation.

Activation of the HGF/MET signaling axis is known to correlate with cancer progression [1]. Therefore, MET-targeted therapy has been employed in various cancers, including non-small-cell lung cancer (NSCLC) and metastatic medullary thyroid cancer, and acceptable outcomes have been reported [10,11,18,25,26,27,28]. In the treatment of NSCLC, the amplification of MET induces resistance toward the TKI targeting the epidermal growth factor receptor (EGFR), and combined therapy of EGFR-TKI and MET-TKI has shown significant efficacy [28]. On the other hand, acquired resistance toward MET-targeted therapy has been reported as a serious issue [29,30]. Cancer-induced utilization of an alternative signaling pathway, the exon 14 skip mutation of MET, and mutation-induced MET amplification were considered as the mechanisms [29,31]. In addition, it has been reported that an HGF-enriched condition significantly reduced the therapeutic potential of MET-TKI in MET-amplified NSCLC and gastric cancer, and additional inhibition of HGF overcame the resistance [32]. Combined HGF and MET-targeted therapy was also reported by Basilico et al. [33]. In their study, a specific anti-MET antibody was used for the inhibition of MET, and a decoy soluble domain of MET was used for HGF binding. Combined therapy resulted in a significant therapeutic effect for pancreatic carcinoma orthotopically transplanted into SCID mice engineered to express human HGF. In the current study, we used capmatinib as an inhibitor of MET (MET-I) and SRI-31215 as an inhibitor of HGF activation (HGFA-I), which is a small-molecule inhibitor of HGFA, hepsin, and matriptase [34]. As a result, combined therapy with MET-I and HGFA-I inhibited the phosphorylation of MET, cell proliferation, and motility of two RCC cell lines. Combined therapy was significantly stronger than a single use of MET-I and HGFA-I in the presence of HGF. In the wound-healing assay, attenuation of the MET-I-induced therapeutic effect was observed with the addition of HGF; however, additional HGFA-I overcame resistance. The phenomenon was observed in both cell lines. Although HGF-independent autophosphorylation of MET was observed in caki-1, additional HGF upregulated proliferation and migration, and co-administration of HGFA-I with MET-I was effective. Joffre reported that the presence of an activating mutation of MET in cancer cells would enhance oncogenic signal activity in the presence of HGF [35]. Therefore, the importance of combined inhibition of HGF activation and MET was suggested for mRCC treatment in an HGF-enriched condition. Control of drug-induced adverse events (AEs) is important in cancer treatment. As common AEs of capmatinib, peripheral edema, nausea, vomiting, and an increased blood creatinine level have been reported [25]. There have been no reports of SRI-31215 administered to humans; however, acceptable safety features in animal studies have been reported [34]. From this point of view, the frequency of dose-dependent AEs may be reduced by a combination therapy compared with single-use dosages while achieving the same effect.

## 5. Conclusions

The expression of MET was increased in both the primary tumor and metastatic sites; however, phosphorylation was significantly upregulated in metastasis compared with the primary tumor. As a treatment, combined therapy of MET-I and HGFA-I showed the strongest inhibition of the phosphorylation of MET and reduced the proliferation and migration of RCC cells in the presence of pro-HGF. The importance of a combined therapy is suggested in treatment of metastatic RCC.

## Figures and Tables

**Table 1:** Patient characteristics.

Age at Primary Treatment (Range)	71 (39–86)
Gender	
Male	22
Female	9
pT stage	
T1	3
T2	2
T3a	18 *
T3b	5
T4	4

* A case is bilateral RCC. pT stage: pathological T stage.

**Table 2:** Expression and phosphorylation of MET in mRCC.

	MET			p-MET		
pT Stage	+	−	*p* Value	+	−	*p* Value
≤T2	4	1	0.9584	0	5	0.8540
≥T3	25	2		4	23	
Site						
primary	29	3	0.6253	4	28	0.0001
metastasis	37	7		31	13	

p-MET: phosphorylation of MET, mRCC: metastatic renal cell carcinoma. Significance was determined by χ^2^ test.

**Table 3:** Expression and phosphorylation of MET in metastasis.

Metastatic Site	MET-Positive	p-MET-Positive	Total
Lung	12	9	14
Bone	6	6	8
Lymph node	5	3	5
Subcutaneous	5	5	5
Adrenal gland	1	1	3
Liver	1	1	2
Pancreas	2	1	2
Retroperitoneum	2	2	2
Brain	1	1	1
Ureter	1	1	1
Omentum	1	1	1

## Data Availability

The data presented in this study are available on request from the corresponding author to protect participants’ privacy.

## Supplementary Materials

The following supporting information can be downloaded at: https://www.mdpi.com/article/10.3390/biomedicines13040811/s1, Figure S1: Kaplan-Meier analysis for OS, primary tumor. Figure S2: Kaplan-Meier analysis for OS, metastasis. Figure S3: Univariable and multivariable analysis of factors associated with OS. Figure S4: Expression and phosphorylation of downstream molecules of MET signaling. Table S1: Expression and phosphorylation of MET in mRCC. Table S2: Comparative immunoreactivity of MET and p-MET in metastases. Table S3: Comparative immunoreactivity of MET and p-MET in metastases (detail).

## Abbreviations

The following abbreviations are used in this manuscript:

MET	HGF receptor
HGF	Hepatocyte growth factor
Pro-HGF	HGF propeptide
HGFA	HGF activator
RCC	Renal cell carcinoma
mRCC	Metastatic renal cell carcinoma
TKI	Tyrosine kinase inhibitor
MET-I	MET inhibitor
HGFA-I	HGF-activator inhibitor
VEGFR	Vascular endothelial growth factor receptor
EGFR	Epidermal growth factor receptor
NSCLC	Non-small-cell lung cancer
*HPN*	Hepsin-coding gene
*ST-14*	Matriptase-coding gene
MAPK	Mitogen-activated protein kinase
CAF	Cancer-associated fibroblast
