# Supplementary material for: Phosphorylation of MET Is Upregulated in Metastatic Sites of Renal Cell Carcinoma: Possible Role of MET and Hepatocyte Growth Factor Activation-Targeted Combined Therapy
Source: Biomedicines. 2025 Mar 28;13(4):811. doi: 10.3390/biomedicines13040811 (PMC12024609; doi:10.3390/biomedicines13040811)
Supplement: Supplementary file 1 [file biomedicines-13-00811-s001.zip › (NC'd MN) Supplemental tables, RCC.pdf]

Supplemental Table 1. Expression and phosphorylation of MET in mRCC.

	MET				p-MET			
**site**	**-**	**+**	**2+**	**p-value**	**-**	**+**	**2+**	**p-value**
primary	3	24	5	P=0.01138	28	4	0	P<0.01
metastasis	7	18	19		13	13	18	

Staining of >50%, strongly positive (2+); 10-50%, positive (+); <10%, negative (-). p-MET: phosphorylation of MET, mRCC: metastatic renal cell carcinoma. Significance was determined by χ^2 test.

Supplemental Table 2. Comparative immunoreactivity of MET and p-MET in metastases

	MET			p-MET		
**metastatic site**	**-**	**+**	**2+**	**-**	**+**	**2+**
Lung	2	6	6	5	5	4
Bone	2	4	2	2	2	4
Lymph node	0	2	3	2	2	1
Subcutaneous	0	1	4	0	0	5
Adrenal gland	2	1	0	2	0	1
Liver	1	1	0	1	1	0
Pancreas	0	2	0	1	1	0
Retroperitoneum	0	1	1	0	1	1
Brain	0	0	1	0	0	1

Staining of >50%, strongly positive (2+); 10-50%, positive (+); <10%, negative (-). p-MET: phosphorylation of MET.

Supplemental Table 3. Comparative immunoreactivity of MET and p-MET in metastases (detail)

patient No.	site	MET	p-MET
1	primary	+	-
1	liver	+	+
2	bone	+	-
2	primary	+	+
3	primary	+	-
3	bone	-	-
4	primary	-	-
4	lung	2+	+

5	primary	+	-
5	lung	+	+
6	primary	+	-
6	adrenal gland	-	-
7	primary	+	-
7	bone	2+	2+
7	subcutaneous	2+	2+
7	subcutaneous	2+	2+
8	primary	+	-
8	lung	+	+
8	retroperitoneum	2+	2+
8	bone	-	+
8	lung	+	2+
9	primary	+	-
9	lung	2+	2+
10	primary	+	-
10	lymph node	+	-
10	lung	+	-
11	primary	-	-
11	liver	-	-
11	adrenal gland	-	-
12	lung	-	-
12	primary	+	-
13	primary	2+	-
13	lung	+	-
14	primary	+	-
14	lymph node	2+	-
15	primary	+	-
15	bone	+	2+
15	bone	+	2+
16	primary	+	-
16	lymph node	+	+
17	primary	+	-
17	bone	2+	2+
17	lung	2+	+
18	primary	+	-

18	lung	+	-
19	primary	+	-
19	subcutaneous	+	2+
19	subcutaneous	2+	2+
19	subcutaneous	2+	2+
20	primary	+	-
20	pancreas	+	+
21	primary	+	-
21	retroperitoneum	+	+
22	primary	+	-
22	ureter	2+	2+
22	omentum	2+	+
23	primary	+	-
23	bone	+	+
24	primary	2+	-
24	lymph node	2+	2+
24	brain	2+	2+
25	primary	-	-
25	lung	-	-
26	primary	+	-
26	lymph node	2+	+
27	primary	+	-
27	primary	2+	+
27	pancreas	+	-
28	primary	2+	-
28	lung	2+	2+
29	primary	+	+
29	lung	2+	+
30	primary	2+	+
30	lung	2+	2+
31	primary	+	-
31	adrenal gland	+	2+

Staining of >50%, strongly positive (2+); 10-50%, positive (+); <10%, negative (-). p-MET: phosphorylation of MET.
