# Supplementary material for: Phosphorylation of MET Is Upregulated in Metastatic Sites of Renal Cell Carcinoma: Possible Role of MET and Hepatocyte Growth Factor Activation-Targeted Combined Therapy
Source: Biomedicines. 2025 Mar 28;13(4):811. doi: 10.3390/biomedicines13040811 (PMC12024609; doi:10.3390/biomedicines13040811)
Supplement: Supplementary file 1 [file biomedicines-13-00811-s001.zip › Supplemental Figure 2-4.pdf]

Supplemental Figure 2

(A)

p-MET	number	OS (month)	95%CI (month)	P-value
Low	28	NA	35-NA	0.955
High	3	NA	10-NA	

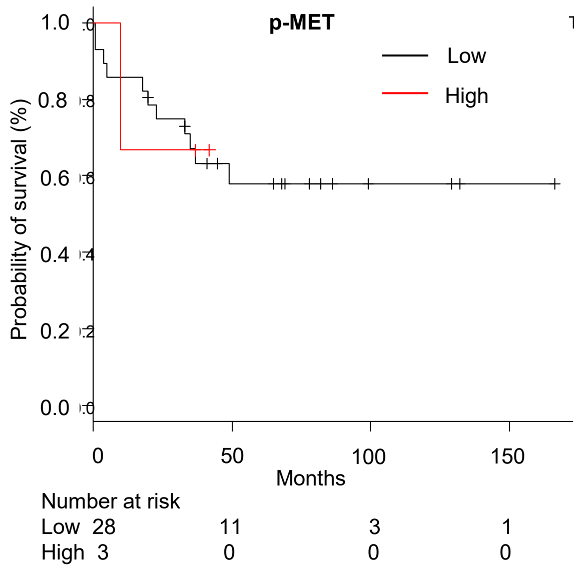

Kaplan-Meier analysis for OS after surgery. RCC patients with low (-) and high (+ and 2+) expression of p-MET at primary site were compared. p value was calculated by log-rank test.

(B)

MET	number	OS (month)	95%CI (month)	P-value
Low	3	NA	20-NA	0.853
High	28	NA	35-NA	

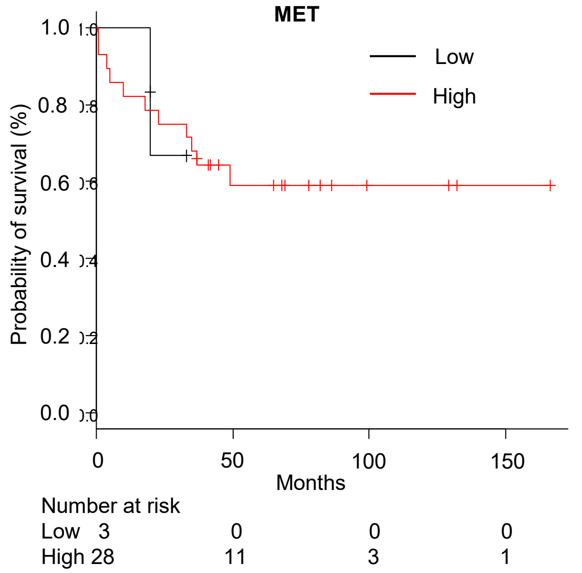

Kaplan-Meier analysis for OS after surgery. RCC patients with low (-) and high (+ and 2+) expression of MET at primary site were compared. p value was calculated by log-rank test.

Supplemental Figure 3

(A)

p-MET	number	OS (month)	95%CI (month)	P-value
Low	11	42	5-NA	0.224
High	20	NA	27-NA	

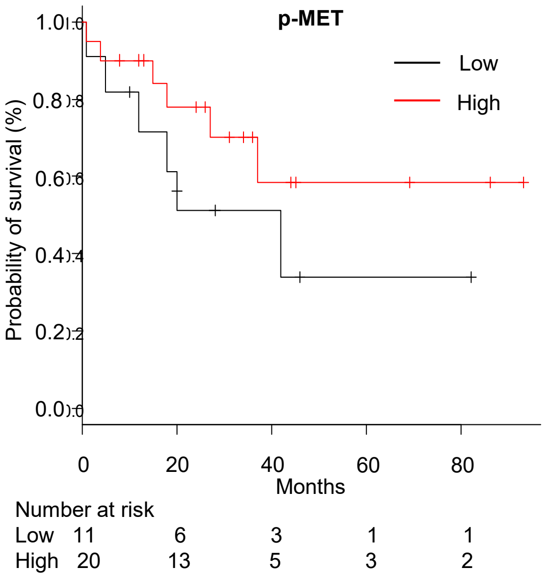

Kaplan-Meier analysis for OS after surgery. RCC patients with low (-) and high (+ and 2+) expression of p-MET at metastasis were compared. p value was calculated by log-rank test.

(B)

p-MET	number	OS (month)	95%CI (month)	P-value
Low	5	20	1-NA	0.0341
High	26	NA	27-NA	

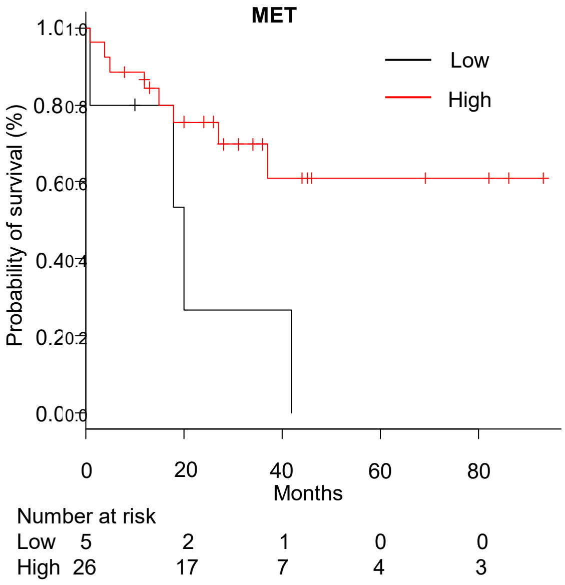

Kaplan-Meier analysis for OS after surgery. RCC patients with low (-) and high (+ and 2+) expression of MET at metastasis were compared. p value was calculated by log-rank test.

Supplemental Figure 4

Variable	Number	Univariable			Multivariable		
		OS(month)	95%CI	*P*-value	HR	95%CI	*P*-value
**p-MET**							
**Negative**	11	42	5-NA	0.22	0.65	0.17-2.44	0.52
**positive**	20	NA	27-NA				
**IMDC risk**							
**Low**	6	NA	NA-NA	**0.0497**	24.9	1.89-329.1	**0.01**
**Intermediate**	17	37	18-NA				
**High**	8	17.5	1-NA				
**KPS**							
**≧80**	23	NA	18-NA	1.00	0.13	0.01-1.48	0.10
**<80**	8	37	12-NA				
**Hb**							
**Normal - high**	14	42	18-NA	0.99	0.23	0.05-1.04	0.06
**Low**	17	NA	15-NA				
**Platelet**							
**Normal – low**	26	NA	27-NA	0.25	0.21	0.01-4.62	0.33
**high**	5	20	1-NA				
**Neutrophil**							
**Normal – low**	22	NA	27-NA	0.0502	0.95	0.09-9.74	0.97
**high**	9	20	1-NA				
**Ca**							
**Normal – low**	25	NA	27-NA	0.20	0.64	0.06-7.43	0.72
**high**	6	20	4-NA				

Univariable and multivariable analysis of factors associated with OS. Statistically significant values are indicated in bold. IMDC: International Metastatic RCC Database Consortium, KPS: Karnofsky Performance Status, Hb: hemoglobin
