# Supplementary figures and images for: Phosphorylation of MET Is Upregulated in Metastatic Sites of Renal Cell Carcinoma: Possible Role of MET and Hepatocyte Growth Factor Activation-Targeted Combined Therapy
Source: Biomedicines. 2025 Mar 28;13(4):811. doi: 10.3390/biomedicines13040811 (PMC12024609; doi:10.3390/biomedicines13040811)

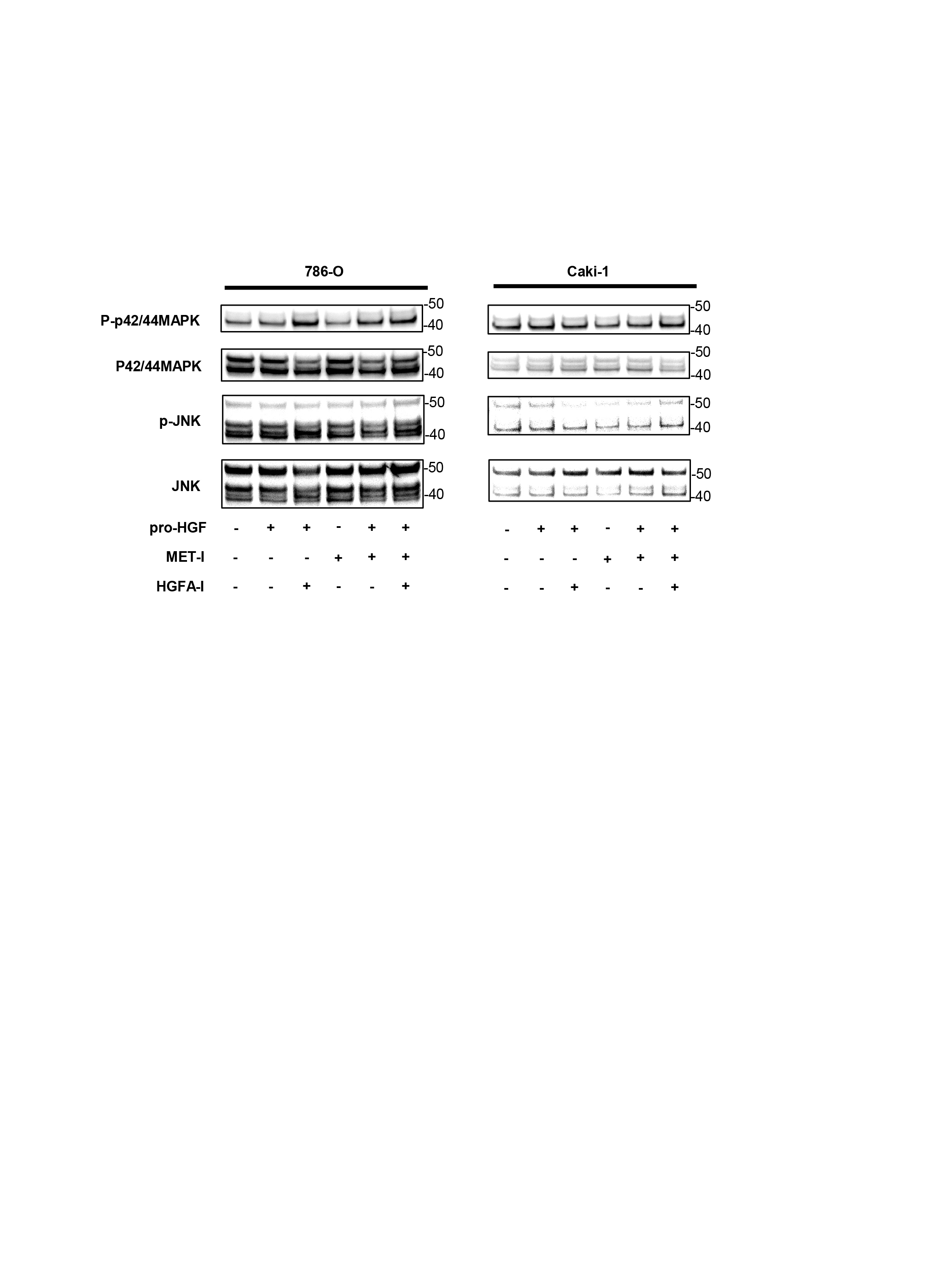

Supplement: Supplementary file 1 [file biomedicines-13-00811-s001.zip › Supplemental Figure 1.jpg]
